# The Government and the Voluntary Hospitals

**Published:** 1920-01-03

**Authors:** 


### THE GOVERNMENT AND THE VOLUNTARY HOSPITALS.

Much misapprehension prevailed until a fortnight ago on questions relating to the suggested nationalisation by the Government of the voluntary hospitals put forward by a few persons devoid of authority. Once the question had been thus raised it was of the first importance that the Prime Minister or the Minister of Health, or both, should take an opportunity to reassure the great body of voluntary hospital supporters throughout the country that the substitution of rate-supported hospitals for the voluntary system was neither feasible nor contemplated. This assurance has now been given on behalf of the Government by Dr. Addison, the Minister of Health. Dr. Addison has endeavoured to clear up the points in the answers given to four questions dealt with in the House of Commons on October 28, November 20, December 11, and December 22, which we publish as they have been received from the Ministry of Health. It will be observed that what Dr. Addison, as Minister of Health, has declared on behalf of the Government is:—

(1) That it would not be within the power of the Ministry of Health, without legislation, to convert hospitals hitherto maintained by voluntary contributions into institutions partly or wholly assisted from public funds. This step would be *ultra vires* in respect of any hospitals not willing to receive such subsidies.

(2) On December 11 Dr. Addison stated that His Majesty's Government did not intend to take over the maintenance and administration of British hospitals.

(3) On December 22 Dr. Addison, in reply to Mr. L. Lyle, stated that the Government had no intention to nationalise the great voluntary hospitals.

### Questions and Answers in the House of Commons.

The following are the verbatim reports of the questions put and the answers received in the House of Commons on the dates already enumerated above:—

### HOUSE OF COMMONS,
**Question and Answer (28th October, 1919).**

Sir A. Warren asked the Prime Minister whether, having regard to the importance of maintaining our hospitals (at present supported by voluntary aid) in full operation, and seeing that it is impossible to raise sufficient funds by begging for their proper upkeep, maintenance, and administration, he will say what steps he proposes taking to arrange for their being taken over by the State and properly provided for?

Dr. Addison: I am aware of the increasing difficulties of many of the voluntary hospitals, and recognise that they affect the proper provision of various forms of medical services in hospitals and in centres or clinics and otherwise; but I am not yet in a position to make a statement thereon.

### HOUSE OF COMMONS,
**Question and Answer (20th November, 1919).**

Hospitals (Maintenance).

Colonel Newman asked the Minister of Health whether the Government have any intention of converting the hospitals hitherto maintained by voluntary contributions into institutions partially or wholly assisted from public funds?

Dr. Addison: The step suggested in the question would not be within the power of the Ministry of Health, without legislation, in respect of any hospitals not willing to receive such subsidies.

### HOUSE OF COMMONS,

**Question and Answer (11th December, 1919).**

Public Hospitals.

Brigadier-General Colvin asked the Minister of Health whether His Majesty's Government intend to take over the maintenance and administration of public hospitals; and whether, in the event of their doing so, cottage hospitals will be excluded?

Dr. Addison: The answer to both parts of the question is in the negative.

### Extract from PARLIAMENTARY DEBATES—HOUSE OF COMMONS,
**Monday, 22nd December, 1919.**

(Pages 390-391.)

Voluntary Hospitals.

Mr. L. Lyle asked the Prime Minister whether he could state on behalf of the Government that there would be no attempt on their part to nationalise the great voluntary hospitals; and whether he was aware that many such institutions were suffering at the present moment on account of charitable people holding back owing to uncertainty as to the Government's intentions?

The Minister of Health (Dr. Addison): Yes, Sir, I can. As I have already stated in reply to a question on the 11th instant, the Government have no such intention.

